# The Multiple Influences of Natural Farming Environment on the Cultured Population Behavior of Kuruma Prawn, *Penaeus japonicus*

**DOI:** 10.3390/ani12233383

**Published:** 2022-12-01

**Authors:** Wenzhi Cheng, Heqian Zhang, Panpan Wang, Yiming Wei, Chuanxi Chen, Yiling Hou, Xiaojie Deng, Siqi Li, Shengyao Sun, Qisi Cai, Yong Mao, Xiangrong Liu

**Affiliations:** 1 Department of Computer Science, Xiamen University, Xiamen 361102, China; 2 National Observation and Research Station for the Taiwan Strait Marine Ecosystem, Zhangzhou 363400, China; 3 Center for Biological Science and Technology, Advanced Institute of Natural Sciences, Beijing Normal University, Zhuhai 519087, China; 4 College of Education for the Future, Beijing Normal University, Zhuhai 519087, China; 5 Jiangsu Key Laboratory of Marine Bioresources and Environment, Jiangsu Key Laboratory of Marine Biotechnology, Jiangsu Ocean University, Lianyungang 222005, China; 6 State Key Laboratory of Marine Environmental Science, Xiamen University, Xiamen 361102, China; 7 College of Ocean and Earth Sciences, Xiamen University, Xiamen 361102, China

**Keywords:** shrimp, behavior, sex ratio, water quality, light intensity, density

## Abstract

**Simple Summary:**

To inform the management of shrimp culture strategies, it is important to uncover the interaction of complex factors with shrimp behaviors in the culture environment. This study was carried out with the objective to investigate the behavioral patterns of *Penaeus japonicus* and the effects of the natural farming environment on its behavior. The results revealed the preference for maintaining silence for *P. japonicus* in their breeding environment, of which the behavior patterns were prominently influenced by the sex ratio. Light intensity is demonstrated as the most influential factor in the behavior of *P. japonicus*, followed by population density. In conclusion, this study provides scientific evidence for shrimp farming of significant practical value.

**Abstract:**

Recent years have witnessed a tremendous development in shrimp farming around the world, which, however, has raised a variety of issues, possibly due to a lack of knowledge of shrimp behavior in farms. This study focused on the relationship between shrimp behavior and the various factors of natural farming environment through situ surveys, as distinguished from the majority of laboratory studies on shrimp behavior. In the survey, the behaviors of kuruma prawn (*Penaeus japonicus*) were investigated in the groups of swimming in the water, crawling on the sand, resting on the sand, and hiding in the sand, followed by the quantification of the sex ratio, water quality, density, and light intensity. The results showed the average proportions of resting, hiding, crawling, and swimming activities of 69.87%, 20.85%, 8.24%, and 1.04%, respectively, of *P. japonicus*. The behavior of hiding, resting, and crawling is significantly affected by the sex ratio of the shrimp (*p* < 0.05). The proportions of hiding behavior exhibited a negative connection with density and a positive connection with light intensity, while the proportions of resting behavior showed the opposite according to both Pearson correlation analysis and multiple linear regression analysis. The light intensity was the only factor that significantly influenced the swimming behavior, in which the probability of the swimming behavior was reduced from 48% to 5% when light intensity varied from 0 to 10 lx, as determined by the generalized linear model. It could be speculated that *P. japonicus* prefers a tranquil environment. Female shrimp might exhibit less aggression and more adventure compared to male shrimp. The findings suggested light intensity, followed by density, as the most crucial element influencing the behavior of *P. japonicus* in the culture environment. These findings will contribute to the comprehension of the behavior of *P. japonicus* and provide a novel perspective for the formulation of its culture management strategy.

## 1. Introduction

The production of crustaceans through aquaculture has become a global industry with significant commercial and economic validity [1]. Up to 2020, shrimps and prawns accounted for almost 16.4% of the total value of internationally exported aquatic products [2]. As one of the world’s most rapidly growing aquaculture sectors, shrimp production is of great significance for many countries [3,4,5]. One of the most common commercial shrimp species is the kuruma prawn, *Penaeus japonicus*, which occupies certain cultivation in China, Australia, the Philippines, and Japan [6,7,8]. The culture of this species is generally performed extensively in growth ponds and has developed into highly intensive indoor culture systems to meet the growing global demand [9,10]. The intensification and densification of shrimp farming have raised increasing problems, involving disease [11,12,13], water pollution [14,15], and individual escapes [16,17], which resulted in drastically decreased aquaculture stocks over the course of a decade. As studies have demonstrated, a comprehensive understanding of the behavior and habits of shrimp may contribute to identifying the countermeasures of the aforementioned problems in aquaculture management [18].

Shrimp behavior is affected by a variety of factors, which could be grouped into three effects: Individual-level effects, water quality effects, and environmental effects [18]. Individual-level effects refer to a variety of factors, including sex [19,20], molting [21], starvation [22], and personality [23], among which sex is important in the behavior of shrimp. Despite the lack of studies on penaeids that link behavioral variations to sexual differences, evidence has demonstrated that males in *Litopenaeus vannamei*, which typically spend the most time on the tank’s bottom, show more energetic swimming behaviors compared to females [20]. In addition, in terms of other crustaceans, such as rock shrimp [24] and American lobster [25], males exhibited higher levels of aggression and feeding activity. Additionally, the sex ratio of shrimp plays a crucial role in the research pm shrimp population biology [26], shrimp evolutionary biology [27], and shrimp behavioral ecology [28]. The sex ratio as a biological factor can influence mating in captivity in penaeid shrimp [28]. However, studies on the relationship between sex ratios and the locomotor behavior of shrimp in farming environments are lacking.

Water quality is one of the most crucial and fundamental factors in the culture of marine shrimp [29,30], which exerts influences mainly concentrated on molting [31,32,33], feeding [17,34], and antennular flicking [35,36]. Numerous studies have demonstrated the high correlation of prawn behavior with water quality such as salinity [37,38], temperature [34,39], pH [40,41], and DO [42,43]. However, the related studies have merely been reported. In the present study, four water quality factors, pH, dissolved oxygen, salinity, and temperature, were taken for examination of their potential influence on the behavior of shrimp.

The environmental effects cover environmental enrichment, stocking density, and the photoperiod [18]. As a benthic organism, shrimps are confined primarily by two-dimensional space but not three-dimensional volume [44]. As a result, the substrate at the bottom of shrimp farms can be considered a significant factor [18]. Previous researchers have demonstrated significant differences in shrimp distribution between tanks with and without artificial substrates [45]. *P. japonicus* represent burrowing benthic animals [46], and the same artificial substrate covered the bottom of the pools in the present study. In this study on the effect of environmental conditions on shrimp behavior, only two variables, stocking density and photoperiod, were evaluated.

The stocking density is another critical aspect of aquaculture production [47,48] and is linked to one of the environmental effects on shrimp behavior [18]. Shrimp development and survival will be impacted by the increased unfavorable behavior in high-density environments, such as cannibalism [45]. Previous work on the effects of varied shrimp stocking densities on shrimp behavior is limited. In *L. vannamei,* it was found that groups with various densities demonstrated variations in their individual behaviors [49]. *Macrobrachium rosenbergii* juveniles showed noticeably more frequent inactivity at a high density compared to at a low density [50]. Another study demonstrated the increased agonistic behavior of *M. japonicus* as the stocking density increased [51].

Another prominent environmental factor is light conditions, with vital significance on both behavior and physiology in the aquaculture of crustaceans [52,53,54]. According to previous studies [55,56], light conditions may be the most determinative element in the locomotor activity of shrimp. For instance, *M. japonicus* is nocturnally active, and captive animals are typically fed after sunset [57], which is closely correlated to the intensity of light available. According to certain research, *L. vannamei*’s feeding behavior can be promoted by light [58], and their motility appears to be reduced by high light compared to weak light [29].

Most studies on the behavior of prawns have been carried out in laboratories and concentrated on a single factor [31,32,33], with a lack of multiple-influence analysis of the natural farming environment on shrimp behavior. In the present study, the sex ratio, dissolved oxygen, pH, salinity, density, and light intensity as individual-level, water quality, and environmental effects on the behavior of *P. japonicus* were quantified. The behavior of *P. japonicus* was divided into four patterns: Swimming in the water, crawling on the sand, resting on the sand, and hiding in the sand. The relationship between multiple factors and behavioral patterns in natural breeding was analyzed. The objective of this study was to examine the behavioral patterns of *P. japonicus* and the effects of factors on that behavior in farms in order to provide managers with a reference for shrimp culture tactics.

## 2. Materials and Methods

### 2.1. Experimental Animals and Ponds

The farm in this study is in Dongshan County, Zhangzhou City, Fujian Province, China. *P. japonicus* cv.‘Minhi1’ aged 9 months old were adopted as the shrimp, with the variety registration number GS-01 004-2014. The prawns were fed once a day, with each feeding constituting 20% of the prawn’s total body weight. Thirty shrimp were randomly chosen for weighing and measuring, which were measured as 95.59 ± 8.38 mm and 10.81 ± 2.26 g, respectively. Female and male shrimps of comparable size were separated into two all-male ponds and two all-female ponds. Before the experiment, the remaining female and male shrimp were placed in three ponds at a ratio of 1:1 and reared for one week. The densities of the three mixing ponds were 12, 12, and 16.6 tail/m^2^, respectively. Two all-male ponds were densified at 16 and 17.6 tail/m^2^, and two all-female ponds had densities of 16.6 tail/m^2^. The seven ponds (2 m × 2.5 m × 1.5 m) were lined with the same grain size (1–1.5 mm) of sand beds (5–8 cm) at the pond bottom. The ponds were replenished with one-third of the water daily using seawater filtration equipment. An oxygen supply was available throughout the experiment.

### 2.2. Survey

The shrimp’s activities were broken down into swimming in the water, scrounging across the sand, lying down on the sand, and hiding in the sand. From 2 June to 9 June 2018, the number of all activities in each pond was recorded at 0:00, 4:00, 8:00, 12:00, 16:00, and 20:00. The Sony RX100M4 camera was utilized to assist in obtaining the number of all shrimps (swimming, crawling, and resting) above the sand. Swimming and crawling shrimps were taken concurrently by two observers three times. The total number of prawns in the pond was available in advance, and the number of prawns hiding in the sand was identified by the total number of prawns in the pool minus the total number of prawns above the sand. The number of shrimps resting on the sand was obtained by subtracting the total number of swimming and crawling shrimps in the water from the total number of shrimps on the sand. The pond water was cleared and aerated without interfering with filming and observation. Each pond’s pH, dissolved oxygen (DO), temperature, light intensity, and salinity were all simultaneously measured. A Bang Bang xiao hei online water quality monitoring device (Bangbang Information Technology Co, Qingdao, China) was adopted to detect the pH and DO, and a HOBO Pendant UA-002-08 Waterproof temperature and light intensity recorder (Onset Computer Corp, Bourne, MA, USA) was used to detect the light intensity and temperature. A Kedid CT-3088 Digital salinometer (AZ instrument Corp, Taichung, China) was used to measure the salinity. The water quality measurements are performed in a fixed corner of the pond, with the equipment placed 0.5 m beneath the water’s surface. Each measurement was repeated three times to obtain an average value. To determine the variation in density, the total number before the experiment and the number of daily fatalities in each pond were recorded.

### 2.3. Statistical Analysis

The multivariate linear model (MLM) [59] and the generalized linear model (GLM) [60] were used to investigate the key factors influencing the behavioral patterns of shrimps in the culture environment, accompanied by the comparison among them. According to the recorded data, the proportion of each behavior type was calculated by dividing the number of shrimps exhibiting each behavior pattern by the total number of shrimps in the pond.

The MLM was established to examine the relationship between crawling, hiding, and resting behavior and water quality, light intensity, and density. The model can be described as:

$$Y=\beta_{0}+\beta_{1}X_{1}+\cdots +\beta_{p}X_{p}+\varepsilon$$

Y represents the percentage of the observed hiding, crawling, and resting behavior, $\beta_{0}\sim \beta_{p}$ for the regression coefficients; and $X_{1}\sim X_{p}$ represents the values of observed water quality, light intensity, and density. The stepwise regression method was taken to optimize the model.

The GLM was established to investigate the effects of water quality, light intensity, and density on the likelihood of swimming behavior occurring. The response variable exhibited a binomial distribution, with 1 denoting the frequent occurrence of swimming behavior and 0 denoting no occurrence. The model used was as follows:

$$log_{e}\left(\frac{\pi }{1-\pi }\right)=\beta_{0}+\sum_{j-1}^{P}\beta_{j}X_{j}$$

$log_{e}\left(\frac{\pi }{1-\pi }\right)$ represents the connection function; $\beta_{0}\sim \beta_{P}$ represents the regression coefficients; and $X_{j}$ is the value of observed water quality, light intensity, and density. P is the probability of swimming behavior.

The Willcox.test was employed to analyze the variations in shrimp behavior during several time periods, and the Wilcoxon signed-rank test was used to compare the differences in shrimp behavior among the all-male, all-female, and mixed ponds. The correlation matrix of pH, temperature, DO, light intensity, salinity density, and the proportion of each behavior type was computed, and the Pearson correlation was obtained to reveal the correlation between the four behavioral patterns and the key influencing factors. R software was used to achieve all of the aforementioned data analysis and modeling [61].

## 3. Results

### 3.1. The Daily Activity and Behavior of P. japonicus

**Figure 1:**
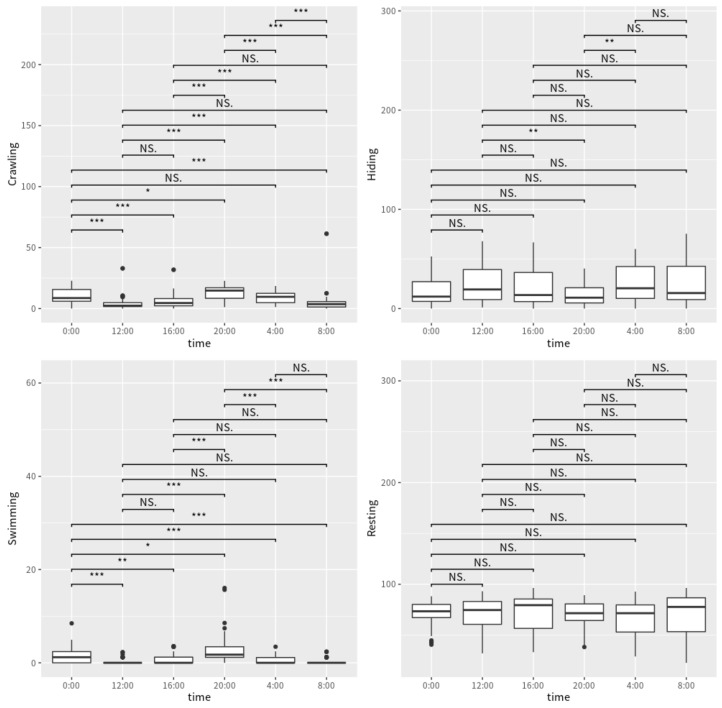
Differences in the proportion of crawling, hiding, swimming, and resting behavior of *P. japonicus* at different time points. Note: NS, represents no significant difference (*p* > 0.05); * represents significant difference (0.005 < *p* < 0.05); ** and *** represent highly significant difference (*p* < 0.005).

Four distinct shrimp behaviors were identified: lying on the sand, creeping on the sand, swimming in the water, and hiding in the sand, among which shrimp resting on the sand occupied the most, with an average of 69.87%; and hiding in the sand and crawling occupied fewer, with averages of 20.85% and 8.24%. Shrimp swimming in the water only occupied an average of 1.04%, making it the least exhibited behavior (Figure 1). With the exception of the time intervals between 0:00 and 4:00, 0:00 and 20:00, 12:00 and 16:00, and 16:00 and 8:00, *P. japonicus* exhibited significant crawling behavior throughout all intervals (*p* < 0.05, Figure 1). The proportion of hiding behavior significantly varied in the groups between 12:00 and 20:00 and between 20:00 and 4:00 (*p* < 0.05, Figure 1). In comparison to all other time points, the proportion of swimming behavior displayed significant differences at 0:00 and 20:00 (*p* < 0.05, Figure 1). No significant difference in the proportion of resting behavior was found at any time point (Figure 1).

### 3.2. The Influence of Sex Ratio on the Behavior of P. japonicus

**Figure 2:**
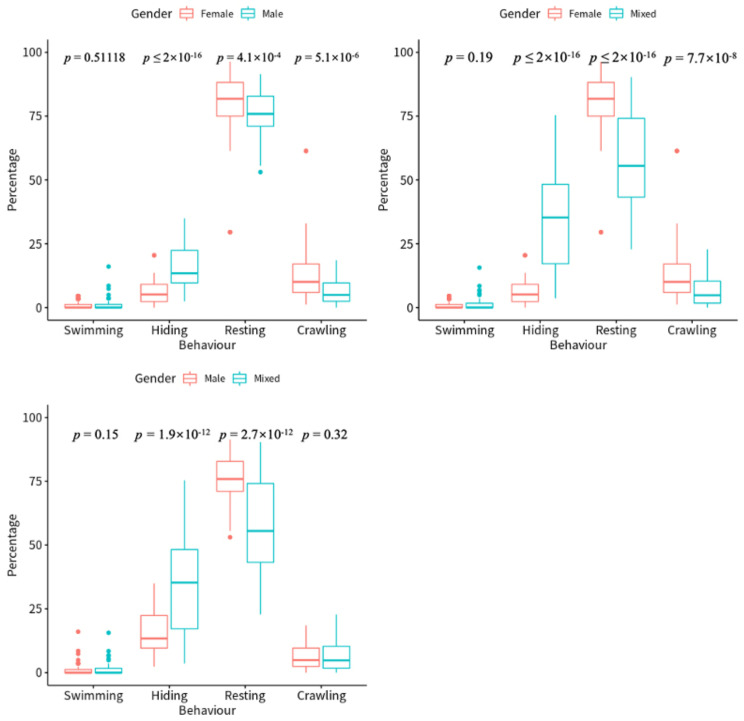
The differences in the proportion of crawling, hiding, swimming, and resting behavior of *P. japonicus* among all-female, all-male, and mixed ponds.

Shrimp resting on the sand had the highest proportion according to each observation in both all-female and all-male ponds, followed by shrimp crawling on the sand surface or hiding in the sand, with the lowest proportion recorded for shrimp swimming in the water (Figure 2). The proportion of shrimp crawling on the sand, hiding in the sand, and resting on the sand significantly varied between all-female and all-male ponds (*p* < 0.05), while shrimp swimming in the water exhibited no sexual differences (*p* > 0.05, Figure 2). A higher prevalence of shrimp crawling on the sand and shrimp resting on the sand was observed in all-female ponds compared to all-male ponds, with proportions of 6.33% and 3.94%, respectively.

Regarding hiding in the sand, the proportion of exclusively female shrimp was 10.29%, which was lower compared to the percentage of exclusively male shrimp. Significant differences in hiding, resting, and crawling behavior were revealed between all-female shrimp ponds and shrimp ponds with a mixture of female and male shrimp (*p* < 0.05, Figure 2). Between the all-male and the mixed shrimp ponds, there were significant differences in hiding and crawling behaviors (*p* < 0.05, Figure 2).

### 3.3. The Influence of Water Quality on the Behavior of P. japonicus

**Figure 3:**
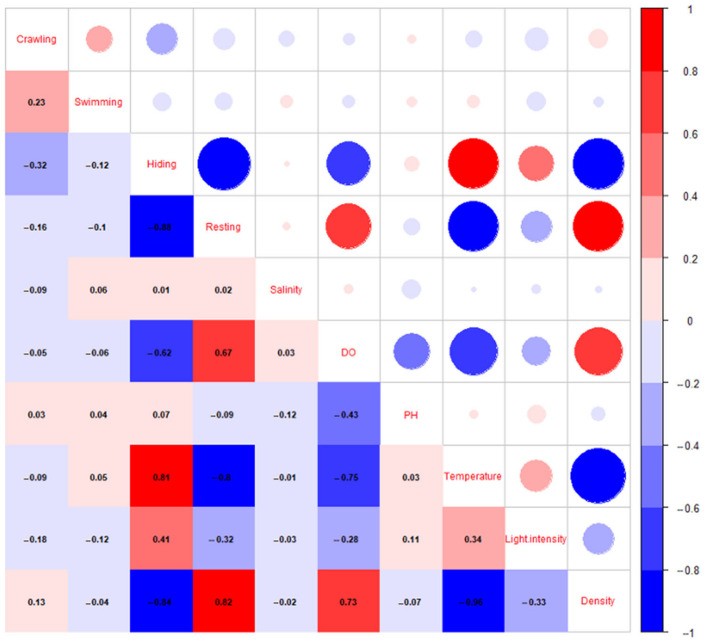
Correlation of shrimp behavior type with water quality, light intensity, and density. Red signifies a positive correlation and blue signifies a negative correlation. The darker the color and the larger the circle, the greater the correlation.

As depicted in Figure 3, no significant correlation was revealed between the salinity and pH and the percentage of crawling, swimming, resting, and swimming behavior. Furthermore, a significant positive correlation of DO with the percentage of resting behavior was displayed, with a correlation coefficient of 0.67 (Figure 3). DO was inversely correlated with the percentage of hiding behavior, with a correlation coefficient of −0.62 (Figure 3). The percentages of crawling and swimming behaviors showed no significant correlation with DO (Figure 3). A significant linear relationship of DO with the percentage of resting behavior was provided in Table 1, with a linear regression coefficient of 2.34. There was a positive correlation between temperature and the percentage of hiding behavior and a negative correlation between temperature and the percentage of resting behavior, with correlation coefficients of 0.81 and −0.8, respectively (Figure 3). The percentages of crawling and swimming behavior showed no significant correlation with temperature (Figure 3).

### 3.4. The Influence of Density on the Behavior of P. japonicus

According to Figure 3, density exhibited a negative correlation with the percentage of hiding behavior and a positive correlation with the percentage of resting behavior, with correlation coefficients of −0.84 and 0.82, respectively. As the analysis of the relationship between density and the percentage of hiding and resting behavior in Table 1 revealed, the coefficients were −5.51 and 5.54, respectively. Density was not significantly correlated with the percentage of crawling or swimming behavior (Figure 3).

### 3.5. The Influence of Light Intensity on the Behavior of P. japonicus

**Figure 4:**
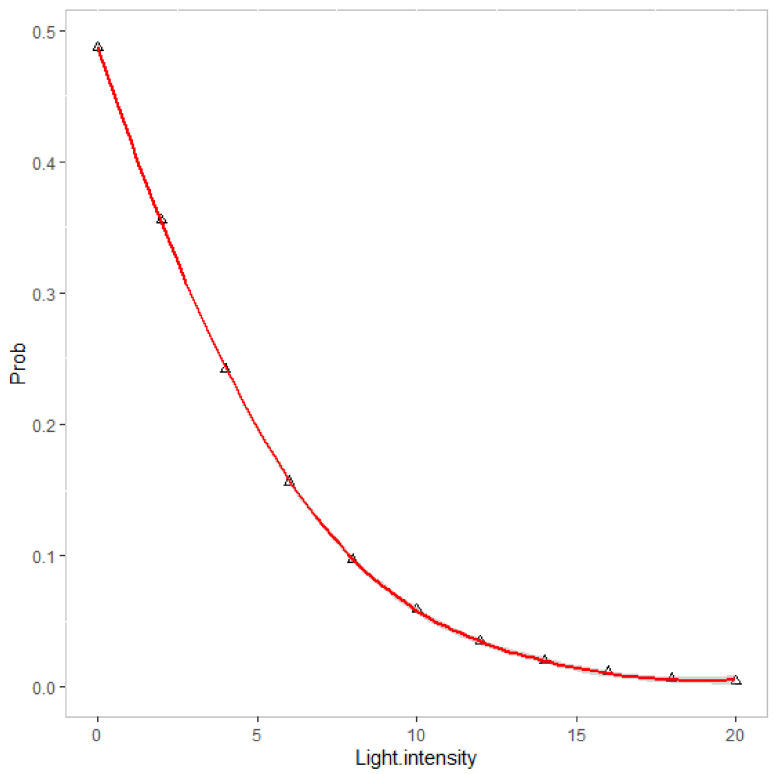
Prediction of the relationship between the probability of swimming behavior and light intensity based on a generalized linear model.

Crawling, swimming, and resting behaviors all showed negative correlations with light intensity (correlation coefficients of −0.18, −0.12, and −0.32), while hiding behavior showed a positive correlation (correlation coefficient of 0.41) (Figure 3). A significant linear relationship between the percentage of hiding with resting behavior was revealed, with the coefficients of linear regression of 1.13 and −0.11, respectively (Table 1). The generalized linear model findings showed a significant relationship between the amount of light and investigation time and the preference for swimming behavior, which showed that the probability of swimming behavior changed from 48.7% to 5% when the light intensity changed from 0 to 10 (Figure 4).

## 4. Discussion

### 4.1. The Daily Activity and Behavior of P. japonicus

This is the first report investigating the behavior of *P. japonicus* in a natural farming environment. It was found that *P. japonicus* exhibited a significant circadian rhythm and a preference for solitude in a sand-filled aquaculture pond. Rather limited studies have categorized the behavior of prawns following their presence, with the majority concentrated on their emergence [62,63]. Shrimp was demonstrated to rest during the day and emerge at night to search for food [52,53,63], which is similar to *P. japonicus*. The behaviors of burrowing shrimp *Callianassa subterranea* were divided into several categories, and resting occupied 33.9 ± 2.4% of the time [64]. Another study showed 40% for resting time and 30% for burrowing time in the burrowing shrimp *Upogebia pusilla* [65]. In the water, an average of 90.45% preferred to hide in the sand or rest on the sand’s surface. On the other hand, it could be speculated that *P. japonicus* was quieter in comparison to other burrowing shrimp species.

### 4.2. The Influence of Sex on the Behavior of P. japonicus

This study demonstrates the significant impact of the ratio of females to males in shrimp ponds on the behavior of *P. japonicus*. Significant differences in the hiding and resting behavior were revealed between mixed and all-female or all-male shrimp ponds, indicating that sex ratios may influence the shrimp community and social behavior patterns, as revealed in resting and submerged sand behavior. Penaeid shrimp females are often larger than males in body size [19,20,66,67] due to a greater gain in mass at each molting cycle [68], as the mixed-sex cultures and male mono-sex cultures both grow more slowly compared to female mono-sex cultures, according to research on *P. monodon* [67,68] and *L. vannamei* [19,20]. In the present study, the resting, concealing, and crawling behaviors of *M. japonicus* considerably differed between all-male and all-female ponds, as all-female shrimp favored lying or crawling on the sand while all-male shrimp favored hiding in it. The following factors could explain the phenomena. Despite the more prominent aggression of male shrimp compared to their female counterparts [18], male shrimp may be more fearful and prefer to penetrate the sand rather than engage in combat with other male shrimp in a pond, as burrowing behavior is a crucial defense mechanism [55]. Female shrimp may be less aggressive but more courageous compared to male shrimp. As a result, there could be more shrimp hiding in the sand in the all-male pond compared to the all-female pond. In addition, due to the increased activity of female shrimp on the surface of the sand compared to the male shrimp, the probability of shrimp capturing food in the male pond was lower. This could explain the lower growing rate of male mono-sex cultures compared to female mono-sex cultures.

### 4.3. The Influence of Water Quality on the Behavior of P. japonicus

Good water quality is essential for the proper function of animals and for shrimp aquaculture to run normally [39]. This research is the first to explore the combined effects of temperature, pH, dissolved oxygen, and salinity on prawn behavior rather than a single water-quality factor. In terms of shrimp resting and concealing behavior in farms, temperature and DO exert a substantial impact, in which a negative link was found between temperature and the percentage of shrimp resting on the sand and a positive link was found between temperature and the percentage of shrimp hiding in the sand, and dissolved oxygen exhibited the reverse pattern. The too-high temperature and low dissolved oxygen imply the tendency of an uncomfortable environment where *P. japonicus* is cultured, which may choose to stay in the sand rather than on the sand’s surface or in the water. The aforementioned findings exert significant guiding relevance for the aquaculture production process but still require further experimental verification. The control of temperature and the concentration of dissolved oxygen throughout the breeding process requires specific attention. As several studies state, crustaceans cultured in low-pH environments exhibit much decreased antennular flicking, which, in turn, reduces the locomotor activity [35,36], and salinity below 10 can increase the locomotor activity of *L. vannamei* juveniles [38]. The varying range of pH and salinity was extremely obscure since this survey adopted the normal range of water quality change in the breeding environment, rather than the severe range considered in other experimental environments. In the present survey, salinity and pH did not significantly affect the behavior, which, however, does not guarantee the absence of this relationship in subsequent experiments. Future research is required to comprehend how pH and salinity affect prawn behavior in farms.

### 4.4. The Influence of Density on the Behavior of P. japonicus

In aquaculture, stocking density is another crucial factor [47,69], which was observed to be negatively related to the percentage of concealing behavior and positively related to the percentage of resting behavior. Decreased swimming behaviors have been observed at lower stocking densities compared to higher stocking densities [18], which is inconsistent with the findings of this work. *M. japonicus* may choose to rest on the sand as a defense strategy, which could explain why shrimp in ponds with higher densities tended to rest on the sand’s surface, where shrimp are more aroused by chemicals, tactile, or visual interference from other people, increasing the pressure exerted on shrimp to obtain food. Prawns can defend themselves effectively on the sand but they are unable to obtain food. They must congregate in the sand to engage in competition for food. The best defense strategy against attacks from other shrimp is to stay stationary on the sand, which could be a peculiar behavior of *M. japonicus* in the cultural setting.

### 4.5. Light Intensity on Behavior of P. japonicus

Our study was in accordance with previous studies’ findings that shrimp locomotor activity was most likely influenced by light conditions, which might be the most critical single factor [55,56]. Light showed the most prominent impact on the occurrence of swimming behavior, in addition to a linear connection with the hiding and resting behavior of shrimp. It is meaningful to investigate the specific causes of this behavior transformation due to light, which may be connected to their biological clock. The nocturnal emergence of *P. duorarum* and *P. attecus* was revealed to be consistent in “dim” light (3 × 10^−5^ lx) and “bright” light (3 × 10^−3^ lx), but the prawns were more active in dim light [70]. The relationship between the preference for swimming behavior and light intensity based on the generalized linear model was predicted to be in line with this trend, despite the failure of quantifying the light intensity of “dim” and “bright” light in this survey. This finding might inspire certain management strategies for shrimp culture. In this study, the modelling for shrimp behavior was carried out only based on light data without eliminating the effect of circadian rhythms on shrimp locomotor behavior. The effect of light intensity on shrimp locomotor behavior is likely to be a reflection of circadian behavior. Further experiments are required to distinguish between the effects of light and rhythm on the locomotor behavior of shrimp.

## 5. Conclusions

*P. japonicus* showed a distinct diurnal rhythm and a preference for tranquility in a sand-filled aquaculture pond. The sex ratio of *P. japonicus* in the pond is a significant factor in altering the behavioral patterns of shrimp. In cultivation, temperature and dissolved oxygen exerted a more prominent influence on shrimp behavior compared to salinity and pH. Density will considerably impact the *P. japonicus* hiding and resting patterns. Light intensity played the most unique and crucial role in shaping *P. japonicus* behavior, which, in turn, significantly influences its resting and hiding behavior, accompanied by the likelihood that it will engage in swimming behavior. The observation of *P. japonicus* in culture ponds reveals the significance of manipulating light intensity and management variations in density in a natural farming environment.

## Figures and Tables

**Table 1:** Results of the model between the behavior and key factors.

Behavior	Fitted Equation	R^2^	*p*
Hiding	Hiding percentage (%) = 103.02 + 1.13 Light.intensity − 5.51 Density	0.77	<0.001
Resting	Resting percentage (%) = −30.78 + 2.34 DO − 0.11 Light.intensity + 5.41 Density	0.74	<0.001
Swimming	ln(P/(1 − P)) = −0.052 − 0.27 Light.intensity	*	<0.001

* shows no significant correlation.

## Data Availability

The data presented in this study are available on request from the corresponding author.
